# Malignancy in Tumors

**Published:** 1879-06

**Authors:** P. W. Van Peyma


BUFFALO

### Medical and Surgical Journal.

VOL. XVIII. JUNE, 1879. NO. 11.

### Original Communications.

:o:

*ART. I.—Malignancy in Tumors* By P. W. VAN PEYMA, M. D.

The term malignancy, as applied to tumors, is understood as denoting a tendency to spread rapidly and to reeur after removal.

It seems to be a quite generally accepted belief, that the boundary lines between benign and malignant growths are sharply drawn and well defined; that, either by the microscope or otherwise, an expert should be able, invariably, to assign to one of the two classes any given pathological growth.

It will be the purpose of this paper to attempt the refutation of this opinion. To endeavor to prove a gradual transition from the benign to the malignant, and conversely; at the same time to show why it is, that, on the one hand, the carcinomatous and sarcomatous growths are so very malignant, while, on the other, the Lipomata and others are scarcely at all so.

The accomplishment of this purpose will necessitate a study of the histological characteristics of the various tumors; and we will therefore devote a few words to this part of our subject.

A simple minute mass of protoplasm, without either nucleus or cell wall, but capable of independent life, is, according to the present view, acknowledged as a cell. But a typical cell, is one possessed of a cell wall or membrane, a nucleus and nucleolus.

According to this definition the human ovum is a typical cell; its vitelline membrane corresponding to the cell wall; the vitellus to cell contents; the germinal vesicle to the nucleus, and the germinal spot to the nucleolus. One of the most marvelous phenomena of nature, is the development of the human body from this single, microscopical cell. The accomplishment of this almost limitless development is the result of segmentation. The single original cell is multiplied into millions. These, during the earliest embryonic life, resemble each other so closely as to be indistinguishable and are called embryonic cells, or, because of their apparent want of any determined future, indifferent cells. Soon, however this similarity gives way to heterogeneity. Of the cells, at first so similar, one elongates to become fusiform; another undergoes corrugation, becoming stellate, &c., &c.

Thus do the indifferent or embryonic cells develope into the cells of epithelium, connective tissue, bone, cartilage, muscle and nerve. This proceeding is termed the process of differentiation.

The cells during early embryonic life arrange themselves into three germinal layers; the middle, termed the hæmoblast or intermediary nutritive apparatus, is intended for the blood vessels. Of the external, called the neuroblasts, and properly the organopoictic, the lower layer furnishes the organs of respiration and digestion; while the upper is the foundation for the organs of motion and sensation. This is as far as the course of the various cells has been definitely and positively followed. Of the various cells of the body, the ones of special interest to us in this connection, are those of the connective tissue and the epithelium; and it will be noticed that they thus early have separate sources; the connective tissue from the middle, the epithelial from the external. This fact seems to argue a wide difference between them.

On the other hand, they are found in most intimate contact throughout the developed body. These two facts, seemingly pointing in opposite directions, have been variously interpreted, and given rise to considerable discussion amongst pathologists.

The main question being, whether epithelium invariably and necessarily arises from epithelium, or, whether it may not and does not occasionally arise from connective tissue cells. The bearing of this question will be more apparent when we come to compare and contrast Carcinoma with Sarcoma.

The next subject requiring consideration is contained in the proposition that normal or healthy growth is the type of the pathological. This is a subject which admits of wide and interesting elaboration, but we must content ourselves with simply pointing out the relation existing between tumors and the normal tissues.

Each tumor has its normal prototype. Of the various formed connective tissues, bone is represented by Osteoma; cartilage by Enchondroma; tendon by Fibroma. Of the higher tissues the Myoma corresponds to muscle; Neuroma to nerve, &c. The unformed connective tissue, that which has not developed into the formed, such as bone, cartilage, &c., is represented by the Sarcoma. And lastly, Epithelium is the prototype of Cancer or Carcinoma.

The difference then between Sarcoma and Carcinoma, is, that they are pathological imitations of different tissues. It is true, that some pathologists have claimed an occasional origin of Cancer from connective tissue; and there is still some reason for thinking that epithelium may possibly, in some rare instances, arise from connective tissue cells. But the belief in the radical difference of the two growths is rapidly gaining ground, and the view just given is the one generally accepted.

Having reviewed the origin of several of the tissues and their relation to the various pathological growths, we turn now to a consideration of the histological appearances of the two pathological new formations which most interest us in this connection, viz: Sarcoma and Cancer.

But before proceeding to this, we must bear in mind the histology of their physiological prototypes,—the connective tissue and epithelium. We recall then, as regards the epithelium, that there are several varieties; such as the pavement, cylindrical or conoidal, &c. Also that it is generally in very intimate relation to the connective tissue, especially is this the case in glandular tissues. Of the connective tissue cells, although many are described, the two principal varieties are the stable or fixed, and the movable or wandering. This latter variety cannot be distinguished from the lencocyt or white blood corpuscles, and undoubtedly has its origin in the blood, passing through the vessel wall by a process termed migration. The stable connective tissue cell presents many varieties, being often spindle-shaped, occasionally stellate, and so on. The microscopical appearance of Osteoma is very similar to that of bone; a Neuroma resembles nerve tissue, &c.

Sarcoma being a pathological growth of connective tissue origin and type, we find several varieties corresponding to the several kinds of connective tissue cells. The following varieties are usually mentioned—the small round celled, the spindle celled, both large and small, the myeloid, a variety in which the cell before dividing enlarges greatly and becomes multinucleated, and lastly, where the tendency to become spindle shaped is carried to the extreme, and regular fibres are formed, we have the Fibroma, whose type is the formed connective tissue, as, for example, Tendon. The cells of Sarcoma are usually arranged in rows or layers between intercellular matter. This intercellular material is "rarely pure connective tissue," but for the most part contains a tolerably large quantity of albuminous,* caseinous or mucinous elements. It may be either "homogenous granular or fibrillar.*

As to the origin of Cancer, I assume that it is invariably an epithelial growth; classing the so-called "connective tissue Cancer" of Wagner with the Sarcomata. Cancer "proceeds from the upper and lower germinal layers, and therefore belongs to the series of new formations of true epithelium." † The opponents of this view, which is that of Thiersch, "hold that the most different tissues are not alone produced by the cells of the ovum, but that this capacity is enjoyed by all, or indeed by most, young cells, especially granulation cells arising from connective tissue, and the colorless blood corpuscles. They therefore seek the demonstration of epithelial or epitheliomal formation in parts of organs which contain no epithelium denoid from the upper or lower germinal layer." ‡

* Wagner, General Pathology. Page 430.

† Wagner, General Pathology. Page 478.

‡ Wagner, Page 486.

In comparing cancer with epithelium, we must make liberal allowance for the fact that in all pathological growths there is a manifest want of perfection; the growth is too rapid to even approximate, in many instances, the normal perfection. In the words of Rindfleisch, "the entire process makes the impression of an over hasty consumption of capital, where the judicious use of the interest would have given a far better result."

"It is anything else than a tissue of ideal quality. The cells instead of being uniform, vary greatly in shape and character, many of them have grown so rapidly that division or segmentation has failed to keep pace with it; and the result is the large multi-nucleated, myeloid or giant cell. Again others degenerate very early, their breaking down resulting in the free nuclei, nearly invariably present."

As to the character of cancer cells, I quote also from Green. "The cells are characterized by their large size, by the diversity of their forms, and by the magnitude and prominence of their nuclei and nucleoli. In size they vary from $\frac{1}{600}$ to $\frac{1}{1500}$ of an inch in diameter, the majority being about five times as large as a red blood corpuscle. They are round, oval, fusiform, polygonal—exhibiting in short every diversity of outline. * * * * The nuclei, which are large and prominent, are round or oval in shape, and contain one or more nucleoli. The nuclei are perhaps most frequently single; two, however, are frequently met with, and in the softer and more rapidly growing cancers, they may be much more numerous. The cells rapidly undergo retrogressive changes, hence they usually contain molecular fat. They are many times exceedingly destructible, so that sometimes more free nuclei than cells are visible. Cells precisely similar to these are met with in other morbid growths, and also in normal tissues. *There is thus no specific "cancer cells."* It is the general character of the cells, together with their mode of distribution in the meshes of a fibroid stroma, that determines the nature of the growth to which they belong. "The appearance presented by these cells grouped within the alveoli of the cancer sometimes closely simulates in the earlier stages of growth that of simple adenoma,"* only here the cells

* Green's Pathology and Morbid Anatomy. Page 143, 144.

are less irregular and more like the normal. It will be noticed that the quotation denies explicitly the existence of a *cancer cell*, that is, a cell characteristic of this growth. On the contrary it is exactly this want of any thing characteristic; the great variety of shape and size and condition, which is to any extent peculiar. Add to this the arrangement in alveoli, formed of connective or fibrous tissue, and we have all that is in any sense characteristic of cancer, from a histological standpoint. And even this is not as much so as could be wished. We have already noticed its resemblance to adenoma. Wagner says, "The alveolar structure of cancer was long regarded as especially characteristic. This, how ever is not the case. Adenoma also, and many sarcamata and cystomata, show an alveolar structure. To draw conclusions from the alveolar texture of new formations, it is always necessary to consider the structure of the mother tissue." * And, in summing up, he concludes as follows: "From these characters it follows that at the present time there are no strict histological peculiarities. At the present time the notion connected with cancer is especially clinical not anatomical." †

In now closing our anatomical description of Sarcoma and Cancer with a short notice of the course and relation of the blood vessels and lymphatics, we are led to remark a very interesting anatomical difference between the two growths; and one having an important bearing in Pathology. In the Sarcoma the vessels are not supported by a stroma, as is the case in Cancer, but ramify amongst the cells of the growth, hence the facility with which these tumors become generally disseminated On the other hand according to Cornil and Ranvier, the lymphatics communicate directly with the alveoli of Cancer. ‡ This explains the tendency of cancer to infect lymphatic glands.

We now come to the subject proper, viz. malignancy. Its definition has already been given:—a tendency to spread rapidly and to recur after removal. The questions now are upon what does this depend, and why are certain growths more malignant than others. From what has preceded our answer may possibly have

* Wagner. Page 480.
† Wagner. Page 501.
‡ Cornil et Ranvier, Histologie Pathologique. Page 175.

been anticipated—that it depends upon the transmission of certain elements, probably cellular, to different parts of the body. This is possible and actually occurs in three ways.

I. Locally by simple extension of the growth.

II. By means of the Lyphatices.

III. By way of the blood-vessels.

"As a general rule the more juice, or cells a growth contains, and the richer it is in blood-vessels and lymphatics, the more quickly will it infect the lymphatic glands and internal organs," * and conversely. In addition to this another point must receive consideration, viz., the difference in the mode of growth of tumors. The proportion of central and peripheral growth is not the same in all tumors. Cancers and Sarcomata are characterized by a predominantly peripheral growth. Other things being equal, a peripheral growing tumor is more malignant than one whose growths is central. The reason of this is obvious. A centrally growing tumor has its active proliferating cells surmounted by a zone, in many instances a capsule of inactive, if not dead material; while in the case of the peripheral growing tumor the active multiplying and infecting cells are at the periphery in immediate contact with the surrounding tissues. The absorption of the elements of the primary growth has, according to Wagner, been demonstrated. He says, "For some cases it has been demonstrated with certainty that cancer masses as a whole and cancer cells especially which are free in the blood-vessels, having been transported hence and deposited in other parts, become the cause of cancerous formations." † He gives similar testimony as to their entrance into the lymphatics. Their mode of action after reaching the part is according to Green "by virtue of an influence on the cells of the tissue where they lodge, which may be termed a spermatic influence, and which is strictly comparable with that of the sperm cell in the ovum." ‡ That is, it excites the cells of the part to a peculiar activity and multiplication. Of course a similar influence is supposed in the local extension of the primary growth. The comparative fre-

---

* Green, Page 107 and 108.

† Wagner, Page 483.

‡ Green, Page 100.

quency and extension of their infection explains the frequent heterology of malignant growths.

While all tumors are therefore probably more or less likely to recur after removal, those are especially so, which are abundant in cells; more particularly the small round cells (this because of their more ready entrance into the vessels); and are richly supplied with blood-vessels and lymphatics.

These factors we have seen to be in a great degree characteristic of Cancer and Sarcoma. The elongated cells being less fitted for absorption, we find the fibroid tumors, recurrent in a lesser degree. The same may be said of the cells of epithelioma. In this connection Wagner says, "especially do so numerous transitions seem really to exist between the so-called benign and malignant new-formations that a fixed limit is at present, and perhaps always will be impossible." *

It will have been noticed that in giving our view upon malignancy, no allusion has been made regarding constitutional predisposition. The question of the comparative importance of local and constitutional causes in the etiology of malignant growths must still be considered open, but the belief in the greater importance of local causes is daily gaining ground, and is even at the present day accepted by the principle pathologists. The most generally accepted view at present, seems to be, that a certain constitutional predisposition probably exerts *some* influence in determining the peculiarly degenerative and destructive changes. This must, however, not be interpreted as implying any *specific* influence; but rather one, admitting of comparison with that low state of vital activity, seen in certain individuals of broken constitutions, where the tendency to a breaking down of tissues is general. In the study of malignancy we are again reminded in nature there are no sudden jumps from one extreme to another, but always a gradual transition. One kind of morbid action gradually merges into an other. In a paper read before the Erie county Medical Society, two or three years ago, I arrived at somewhat similar conclusions. After asserting the gradual transition of

---

* Wagner, Page 501.

hyperœmia into inflammation, and vice versa, I remarked, "In conclusion I will say, to my mind, inflammation is not alone in the fact of its gradual transition into other conditions. My conviction is settled, that, as we progress in our knowledge of the general principles of disease, the application of the 'law' of transition will be found to approach the universal." Examples or illustrations of this law are found on every side. The fact that authors speak of sarcomatous cancers and carcinomatous sarcomas shows that these growths occasionally merge into each other. Rindfleisch speaks of a mixed tumor which he says, "we must leave undecided whether it is to be reckoned with the sarcomas or carcinomas." * The gradual merging of an adenoma or glandular tumor, into carcinoma, has already been referred to. To quote Rindfleisch once more, "certain authors" he says certainly move the idea of adenoma up and down the scale mentioned, in that they now assign it more to hypertrophy, now more to carcinoma; that however a motion up and down of this kind is possible, just proves the existence of this scale." †

Wagner says, "doubtless between adenoma and glandular cell cancer there are found the most manifold transitions." ‡ These he classes under the head Adenoma Carcinomatodes. One of the synonyms of cancer is 'spongoid inflammation;' and Wagner speaking of the two processes says, "Of many cases" (of cancer) "especially of the stomach and mammae, even after careful microscopical examination, it remains doubtful whether they belong here or represent chronic inflammations with strong cicatricial formation, or with simultaneous glandular hypertrophy." §

Rindfleisch considers hard glandular cancer as dependent upon an interstitial inflammation of slow growth; the cellular products of which are metamorphosed into epithelial forms instead of into pus or connective tissue; this in consequence of an epithelial infection * due to neighboring epithelial cells.

Much more might be said upon this subject of transition. And what has been said, should have received elaboration and elucida-

---

* Rindfleisch, Page 164.
† Rindfleisch, Page 158.
‡ Wagner, Page 493.
§ Wagner. Page 495.

tion. But a little thought upon the subject will enable any one to carry this mode of reasoning in various directions, and that to a far reaching degree.

In conclusion let me call attention to one of the practical points intimately connected with the subject.

If the views contained in this paper are correct, any expert to whom we may hereafter carry a specimen for examination, will not say "this growth is malignant or this growth is benignant and harmless." He will rather express his opinion in relative terms, as, for example, "the specimen which I have examined is more or less abundant in cells; their character, as to shape, more or less adapts them for absorption; the arrangement of its blood-vessels and lymphatics is such that they will or will not greatly facilitate absorption and infection of neighboring tissues; the extent of the degeneration and breaking down of cells, and the comparative number of multinucleated cells and the small round cells, to the exclusion of any decided tendency to elongate and develope, prove its more or less rapid growth and destructive power." The consequence will be that we shall watch all morbid growths with a view to their malignancy, being especially fearful of those possessing the above properties in a marked degree. The question will no longer be, is the growth malignant or benign; but to what degree is it malignant, that is, liable to recur, to spread and be destructive.

The main object of the paper has been, by means of an example to call attention to the fact of transition as seen in Pathology.

Many of the positions taken being contrary to the views held by the majority of medical practitioners, more particularly those who have not given the subject any special study, I have felt warranted in making numerous authoritative quotations.

In the opinion of the writer, more attention should be paid to general principles, both in Disease and Therapeutics. The result would be a diminishing amount of superstitious belief in specifics and a growing clearness of vision in matters medical.

---

* Rindfleisch, 167.

